# Effect of Occupational Toxin Exposure on the risk of dementia; Results of the Korean Longitudinal Study on Cognitive Aging and Dementia (KLOSCAD)

**DOI:** 10.1002/alz.088120

**Published:** 2025-01-09

**Authors:** Hak Hyeon Kim, Yvonne van der Bent, Tae Hui Kim, Kyung Phil Kwak, Bong Jo Kim, Shin Gyeom Kim, Jeong Lan Kim, Seok Woo Moon, Joon Hyuk Park, Seung‐Ho Ryu, Jong Chul Yoon, Dong Young Lee, Dong Woo Lee, Seok Bum Lee, Jung Jae Lee, Jin Hyeong Jhoo, Ji Won Han, Ki Woong Kim

**Affiliations:** ^1^ Seoul National University College of Medicine, Seoul Korea, Republic of (South); ^2^ Seoul National University Bundang Hospital, Seongnam Korea, Republic of (South); ^3^ Leiden University Medical Center, Leiden Netherlands; ^4^ Seoul National University College of Natural Sciences, Seoul Korea, Republic of (South); ^5^ Yonsei University Wonju Severance Christian Hospital, Wonju Korea, Republic of (South); ^6^ Dongguk University Gyeongju Hospital, Gyeongju Korea, Republic of (South); ^7^ Gyeongsang National University Hospital, Jinju Korea, Republic of (South); ^8^ Soonchunhyang University Bucheon Hospital, Bucheon Korea, Republic of (South); ^9^ Chungnam National University Hospital, Daejeon, Daejeon Korea, Republic of (South); ^10^ School of Medicine, Konkuk University, Konkuk University Chungju Hospital, Chungju Korea, Republic of (South); ^11^ Jeju national University Hospital, Jeju‐si Korea, Republic of (South); ^12^ School of Medicine, Konkuk University, Konkuk University Medical Center, Seoul Korea, Republic of (South); ^13^ Kyunggi Provincial Hospital for the Elderly, Yongin Korea, Republic of (South); ^14^ Seoul National University Hospital, Seoul Korea, Republic of (South); ^15^ Department of Neuropsychiatry, Seoul National University Hospital, Seoul Korea, Republic of (South); ^16^ Inje University Snaggye Paik Hospital, Seoul Korea, Republic of (South); ^17^ Dankook University Hospital, Cheonan Korea, Republic of (South); ^18^ Kangwon National University, School of Medicine, Chuncheon Korea, Republic of (South)

## Abstract

**Background:**

The global increase in the aging population has led to a corresponding rise in dementia cases. This study explores the potential link between occupational toxin exposure and the incidence of dementia, aiming to identify high‐risk populations.

**Method:**

We analyzed data from 2,485 male participants aged 60 and over in the Korean Longitudinal Study on Cognitive Ageing and Dementia (KLOSCAD). Participants were categorized into no or very low, low and high toxin exposure risk groups based on their lifetime occupation according to the International Standard Classification of Occupations classification. Exposure to the specific hazard type, including carcinogens, skin sensitizers, airway sensitizers, mutagenic substances, reprotoxic agents and biological hazards were identified in relation to their lifetime occupation. We conducted multinomial logistic regression analysis, adjusting for occupation‐related factors, APOE genotype, age, education, health status and lifestyle behaviors, to investigate the relationship between the levels of toxin exposure risk, specific toxin types, and the incidence of mild cognitive impairment (MCI), Alzheimer’s disease (AD) dementia and other dementia.

**Result:**

Compared to the no or very low toxin exposure risk group, participants in the low toxin risk exposure group (odds ratio (OR) 2.24, 95% CI 1.08 – 43.64) and high toxin risk exposure group (OR 2.97, 95% CI 1.31 – 6.74) showed a significantly increased risk of AD dementia. Exposure to carcinogens, skin sensitisers, reprotoxic agents and biological hazards showed a significant association with the risk of incident AD dementia. Among toxin types, skin sensitizers presented the highest OR of 5.48 (95% CI 1.29‐23.31) in multivariable model. No significant associations were found with MCI and other dementia.

**Conclusion:**

Higher risk of lifetime occupational toxin exposure may be associated with an increased risk of AD dementia, showing consistent results across various toxin types, underscoring the need for heightened attention to toxin exposure in AD dementia prevention.

